# Piezo1 in respiratory diseases: mechanotransduction, cell-specific functions, and translational implications

**DOI:** 10.3389/fphys.2026.1885524

**Published:** 2026-07-01

**Authors:** Min Tao, Yiqi Xu, Liyao Bai, Gang Wang, Wenhui Liang, Qianqian Chen, Chu Qin, Haoda Yu

**Affiliations:** 1 Department of Respiratory Medicine, The Affiliated Wuxi People’s Hospital of Nanjing Medical University, Wuxi Medical Center, Nanjing Medical University, Wuxi People’s Hospital, Wuxi, Jiangsu, China; 2 The Affiliated Wuxi People’s Hospital of Nanjing Medical University, Wuxi Medical Center, Nanjing Medical University, Wuxi People’s Hospital, Wuxi, Jiangsu, China

**Keywords:** acute respiratory distress syndrome, mechanotransduction, Piezo1, pulmonary fibrosis, pulmonary hypertension, respiratory disease

## Abstract

Piezo1 is a mechanically activated cation channel that converts membrane tension, shear stress, matrix stiffness, and tissue deformation into calcium-dependent signaling. In the respiratory system, Piezo1 has emerged as a key regulator of epithelial injury, endothelial barrier responses, pulmonary vascular remodeling, fibroblast activation, and immune cell function. This review synthesizes evidence linking Piezo1 to pulmonary fibrosis, pulmonary hypertension, asthma, acute respiratory distress syndrome, respiratory infection, and chronic obstructive pulmonary disease, while distinguishing direct Piezo1 evidence from broader mechanobiology support. Across these conditions, Piezo1 should not be interpreted as uniformly injurious or protective. Its net effect depends on the sensing cell type, force profile, disease phase, and downstream program engaged. We therefore propose a translational framework in which Piezo1-targeted strategies require temporal, regional, and lineage-specific control rather than systemic nonselective activation or inhibition.

## 1 Introduction

Mechanical forces are central to lung biology. Breathing cycles, airway narrowing, blood flow, microvascular pressure, extracellular matrix stiffening, and leukocyte migration continuously expose pulmonary cells to physical stress. These forces are not passive background phenomena; they actively shape epithelial integrity, vascular tone, inflammatory signaling, and tissue remodeling. Mechanotransduction pathways therefore occupy a central position in both respiratory homeostasis and disease (Li et al., 2014; Li et al., 2015; Bhattacharya and Hough, 2019).

Among the major mechanosensors identified to date, Piezo1 has attracted particular interest because it directly couples membrane deformation to cation influx and downstream signaling. Since its discovery, Piezo1 has been implicated in vascular development, endothelial flow sensing, blood pressure regulation, smooth muscle signaling, and immune activation. In the lung, the same channel appears to influence several core disease processes, including alveolar epithelial apoptosis, endothelial permeability, hypoxic pulmonary vasoconstriction, fibroblast activation, and antibacterial host defense (Coste et al., 2010; Li et al., 2014; Ranade et al., 2014; Ge et al., 2015; Li et al., 2015; Bhattacharya and Hough, 2019; Lhomme et al., 2019; Liang et al., 2019; Solis et al., 2019). An important theme emerging from the literature is that rather than acting as a uniformly harmful or protective channel, the biological output of Piezo1 depends on the pulmonary cell population, the type and magnitude of mechanical force, and the phase of disease in which signaling is engaged. This perspective is especially relevant in respiratory disease, where epithelial cells, endothelial cells, pulmonary arterial smooth muscle cells, fibroblasts, neutrophils, macrophages, and innate lymphoid cells occupy distinct mechanical niches (Li et al., 2014; Li et al., 2015; Bhattacharya and Hough, 2019; Solis et al., 2019; Hellings and Steelant, 2020).

**Figure 1:**
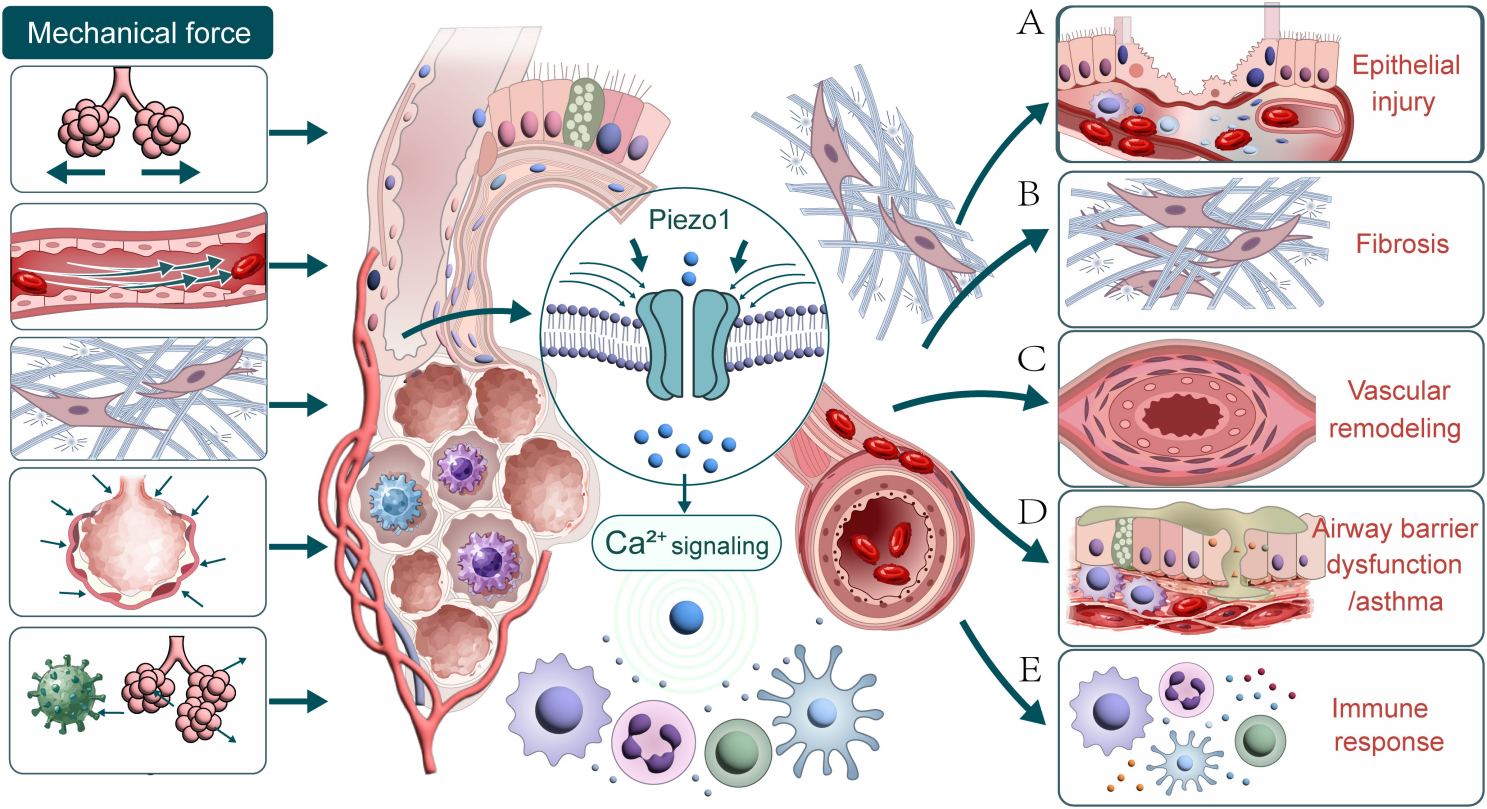
Conceptual framework of Piezo1-mediated mechanotransduction in respiratory disease. Mechanical inputs in the lung, including stretch, shear stress, matrix stiffness, tissue deformation, and inflammatory stress, converge on Piezo1-dependent Ca²^+^ signaling. The downstream effects vary by cell type and disease context: **(A)** epithelial injury and barrier damage; **(B)** fibroblast activation and fibrotic remodeling; **(C)** pulmonary vascular remodeling and vasoreactivity; **(D)** airway epithelial barrier dysfunction and asthma-related remodeling; and **(E)** immune activation, antibacterial defense, and inflammatory tissue injury.

This review synthesizes current PubMed-indexed evidence on Piezo1 in respiratory disease, with emphasis on pulmonary fibrosis, pulmonary hypertension, asthma, acute respiratory distress syndrome or acute lung injury, respiratory infection, and chronic obstructive pulmonary disease. We argue that the translational value of Piezo1 lies in precision modulation rather than global activation or inhibition (Lai and Huang, 2021; Liao et al., 2021; Wang et al., 2021). We define direct Piezo1 evidence as studies that measure Piezo1 expression, localization, activity, or functional consequences after Piezo1 activation, inhibition, silencing, or genetic manipulation in relevant cells or disease models. Broader mechanobiology support refers to studies that define matrix stiffness, stretch, pressure, barrier mechanics, or remodeling programs that are relevant to Piezo1 biology but do not directly test the channel. This distinction is especially important for extracellular matrix and lung mechanics literature, where mechanobiological remodeling can support but not replace direct Piezo1 evidence (Guo et al., 2022; Migulina et al., 2023; Burgess and Gosens, 2024). The overall conceptual framework of Piezo1-mediated mechanotransduction in respiratory disease is illustrated in **Figure 1**.

## 2 Structural and biological basis of Piezo1 signaling in the lung

Piezo1 is a trimeric mechanosensitive ion channel with a curved three-bladed architecture optimized for sensing membrane tension and mechanical deformation. Structural studies showed that each subunit contains numerous transmembrane helices and that the peripheral blades are mechanically coupled to a central ion-conducting pore. This design allows Piezo1 to respond to stretch, shear stress, compression, and substrate stiffness with rapid cation entry, especially calcium influx (Coste et al., 2010; Ge et al., 2015).

Foundational studies further established that Piezo1 is not only a sensor of isolated force pulses but also a physiological integrator of vascular architecture and hemodynamic stress. In endothelial biology, Piezo1 regulates flow-dependent ATP release, vascular development, and blood pressure control, providing a mechanistic template for understanding how the same channel may function in the pulmonary circulation (Li et al., 2014; Ranade et al., 2014; Li et al., 2015).

Within the lung, Piezo1 expression has been documented in alveolar epithelial cells, airway epithelial cells, pulmonary vascular endothelial cells, pulmonary arterial smooth muscle cells, fibroblasts, myeloid cells, and neutrophils. This broad cellular distribution explains why Piezo1 can influence several apparently distinct pulmonary phenotypes, ranging from barrier dysfunction to vascular remodeling and innate defense. The key shared mechanism is calcium entry, but the downstream outputs differ by lineage and microenvironment (Bhattacharya and Hough, 2019; Lhomme et al., 2019; Liang et al., 2019; Solis et al., 2019; Wang et al., 2021; Fang et al., 2023).

Although this review focuses on pulmonary cells, the significance of Piezo channels also comes from their role in neurons and mechanical sensing. Piezo channels, particularly Piezo2 in sensory neurons, provide a canonical example of how mechanical force can be converted into ion flux and sensory perception. This neuronal framework is conceptually useful for mechanotransduction, but current respiratory disease evidence is much stronger for Piezo1 in epithelial, endothelial, smooth muscle, fibroblast, and immune compartments than for Piezo1-dependent signaling in pulmonary neurons (Morris, 1990; Martinac, 2004; Coste et al., 2010; Zhao et al., 2016; Jin et al., 2020; Lewis and Grandl, 2021; Beverley and Levitan, 2024; Xiao, 2024).

In epithelial cells, Piezo1 signaling is commonly associated with apoptosis, ATP release, and junctional disruption. In endothelial cells, Piezo1 may either increase permeability or mediate adaptive vasodilatory and barrier responses, depending on the force profile. In pulmonary arterial smooth muscle cells, Piezo1 supports proliferative and vasoconstrictive programs. In fibroblasts and myofibroblasts, it couples stretch to extracellular matrix production and YAP/TAZ-associated remodeling. In innate immune cells, Piezo1 links mechanical stimulation to inflammatory and antimicrobial functions (Lhomme et al., 2019; Liang et al., 2019; Solis et al., 2019; Liao et al., 2021; Wang et al., 2021; Xu et al., 2022; Fang et al., 2023; Yang et al., 2023; Mukhopadhyay et al., 2024; Zheng et al., 2024).

## 3 Mechanistic axes linking Piezo1 to respiratory pathophysiology

### 3.1 Direct mechanotransduction and calcium signaling

The first major axis is direct mechanotransduction. Mechanical stretch, elevated pressure, cyclic deformation, and matrix stiffening can open Piezo1 and trigger calcium-dependent signaling cascades. In respiratory tissues, these cascades intersect with ATP release, calpain activation, ERK signaling, AKT signaling, nitric oxide pathways, and YAP/TAZ-mediated transcriptional remodeling. Because these pathways regulate survival, junction integrity, metabolism, and matrix production, Piezo1 is positioned at the interface between physical stress and disease progression (Li et al., 2014; Ranade et al., 2014; Li et al., 2015; Lhomme et al., 2019; Liang et al., 2019; Wang et al., 2021; Zheng et al., 2024).

### 3.2 Barrier regulation

The second axis is barrier control. Pulmonary epithelial and endothelial barriers are both mechanically sensitive, and excessive force can convert adaptive mechanosensing into tissue injury. In airway epithelial cells, Piezo1 activation has been linked to degradation of tight junction proteins and increased permeability. In pulmonary endothelial cells, pressure-related Piezo1 signaling may promote hyperpermeability and edema, whereas in other stretch conditions Piezo1 may contribute to endothelial adaptation and vasorelaxation. These apparently divergent findings likely reflect differences in cell type, force direction, force amplitude, and disease timing rather than true contradiction (Lhomme et al., 2019; Liang et al., 2019; Lai and Huang, 2021; Wang et al., 2021; Liu et al., 2024).

### 3.3 Inflammation and mechanical immunity

The third axis is inflammation and mechanical immunity. Piezo1 has emerged as a regulator of how myeloid cells interpret cyclic force, deformation during migration, and mechanically altered tissue environments. In macrophages, Piezo1 supports stiffness sensing and can influence polarization and inflammatory activation, while acute contact between profibrotic macrophages and fibroblasts can mechanically engage Piezo1-dependent fibroblast activation. In neutrophils, Piezo1-dependent mechanosensing during transendothelial migration, shear exposure, or lung stiffness can enhance bactericidal, NETotic, or proangiogenic programs. Recent lymphoid-cell studies further suggest that Piezo1 can restrain or reprogram group 2 innate lymphoid cell activity during airway hyperreactivity and lung inflammation. These studies extend the role of Piezo1 beyond structural sensing and identify it as an active participant in host defense (Solis et al., 2019; Atcha et al., 2021; Baratchi et al., 2024; Ezzo et al., 2024; Hurrell et al., 2024; Mukhopadhyay et al., 2024; Xie and Hang, 2024; Pirri, 2025; Lim et al., 2025; Wang et al., 2025; Wang et al., 2025; Zhao et al., 2025).

### 3.4 Vascular and interstitial remodeling

The fourth axis is tissue remodeling. In pulmonary hypertension, Piezo1 is involved in endothelial signaling, smooth muscle phenotypic switching, vasoreactivity, and right heart-loading pulmonary vascular remodeling. In pulmonary fibrosis, Piezo1 links epithelial injury and fibroblast activation to extracellular matrix deposition. Across these settings, Piezo1 functions as a bridge between force sensing and persistent structural change (Liao et al., 2021; Xu et al., 2022; Fang et al., 2023; Yang et al., 2023; Zheng et al., 2024; Wang et al., 2025).

## 4 Piezo1 in major respiratory diseases

### 4.1 Pulmonary fibrosis

Pulmonary fibrosis is one of the disease settings in which the evidence for Piezo1 is both substantial and directionally consistent. In acute respiratory distress syndrome-related fibrotic progression, mechanical ventilation can aggravate lung fibrosis through Piezo1-mediated epithelial-mesenchymal transition, linking abnormal stretch to calcium signaling, ATP release, and fibrogenic reprogramming. This is important because it places epithelial Piezo1 upstream of a mechanically driven injury-to-fibrosis transition rather than merely downstream of tissue damage (Fang et al., 2023; Liu et al., 2024).

Recent studies have expanded this profibrotic framework beyond the epithelium. In human lung fibroblasts, Piezo channels modulate calcium signaling, ERK activation, and matrix-related responses under mechanical stimulation. In parallel, a 2025 study demonstrated that PIEZO1 in periostin-positive myofibroblasts promotes activation and pulmonary fibrosis in mice. Complementary 2026 work on the matrix-stiffness landscape in idiopathic pulmonary fibrosis further reinforces the view that fibrotic lung tissue should be interpreted as a mechanically active niche rather than a passive scar. Together, these observations support a feed-forward model in which tissue stiffness and cyclic deformation activate Piezo1-relevant pathways, Piezo1 supports profibrotic signaling in susceptible compartments, and extracellular matrix deposition further increases mechanical load (Suki and Bates, 2008; Marinković et al., 2013; Shimbori et al., 2013; Fiore et al., 2015; Júnior et al., 2021; Guo et al., 2022; Trotsyuk et al., 2022; Ezzo et al., 2024; Papavassiliou et al., 2024; Zheng et al., 2024; Xu et al., 2025; Chen et al., 2026).

From a translational perspective, pulmonary fibrosis therefore appears to be one of the strongest candidates for cell-targeted Piezo1 inhibition. However, even in this relatively coherent disease context, the therapeutic question is not simply whether Piezo1 should be blocked. It is more precisely whether epithelial and fibroblast-lineage Piezo1 should be inhibited within defined fibrogenic time windows, especially during the transition from unresolved acute injury to progressive matrix remodeling (Fang et al., 2023; Zheng et al., 2024; Xu et al., 2025).

### 4.2 Pulmonary hypertension

Pulmonary hypertension is the second respiratory disease area with strong evidence implicating Piezo1. Endothelial and smooth muscle compartments both appear to participate, but not necessarily in the same way. In pulmonary arterial endothelial cells, Piezo1 expression is increased in pulmonary hypertension and is linked to mechanosensitive pathways associated with remodeling. In pulmonary arterial smooth muscle cells, Piezo1 promotes phenotypic switching, proliferation, and vasoconstrictive responses relevant to hypoxic pulmonary hypertension (Xiao et al., 2020; Liao et al., 2021; Wang et al., 2021; Xu et al., 2022; Fang et al., 2023; Yang et al., 2023; Mocumbi et al., 2024; Weinstein et al., 2024).

The pulmonary hypertension literature also illustrates why cell-specific interpretation is essential. Endothelial Piezo1 has been associated with flow-dependent or stretch-dependent vasoregulatory effects, including endothelial nitric oxide-related responses and relaxation of intrapulmonary arteries. At the same time, disease-focused studies support a pro-remodeling role for Piezo1 signaling in both endothelial and smooth muscle compartments. These findings suggest that Piezo1 may shift from adaptive vascular sensing in early or physiological contexts to maladaptive remodeling in sustained disease states (Li et al., 2015; Lhomme et al., 2019; Liao et al., 2021; Wang et al., 2021; Guo et al., 2022).

This duality has immediate translational implications. Global Piezo1 inhibition in pulmonary hypertension may suppress maladaptive remodeling but could also interfere with protective endothelial signaling. Future work should therefore define whether endothelial and smooth muscle Piezo1 act sequentially or in parallel and identify the disease stage at which adaptive signaling gives way to pathogenic remodeling (Liao et al., 2021; Wang et al., 2021; Xu et al., 2022; Fang et al., 2023; Yang et al., 2023).

### 4.3 Asthma and airway barrier disease

The current asthma literature supports a meaningful, though still developing, role for Piezo1 in airway barrier dysfunction. A recent study showed that Piezo1 contributes to airway epithelial barrier injury in asthma, consistent with the broader concept that mechanical and inflammatory stress converge on epithelial junctional instability. This is biologically plausible because epithelial barrier failure is a core feature of chronic airway inflammation and remodeling (Lai and Huang, 2021; Liu et al., 2024).

At the same time, the broader asthma field reminds us that structural cell injury and immune regulation do not always move in the same direction. Reviews of epithelial barrier dysfunction in asthma and allergic airway disease indicate that epithelial damage, mechanical stress, and immune amplification are tightly coupled, but they also leave room for lineage-specific counter-regulatory mechanisms. For Piezo1, this means that future asthma studies should not assume a single whole-lung effect. Instead, they should distinguish epithelial injury pathways from immune-cell-specific effects (Sweerus et al., 2017; Gon and Hashimoto, 2018; Hellings and Steelant, 2020; Lai and Huang, 2021; Bradding et al., 2024; Liang et al., 2024; Liu et al., 2024; Liu et al., 2025).

Thus, the most defensible conclusion at present is that Piezo1 is a promising mechanistic contributor to airway epithelial dysfunction, whereas its full role in immune-driven asthma phenotypes remains incompletely defined. Recent ILC2 studies reinforce this need for cell-resolved interpretation, because Piezo1 signaling can restrain airway hyperreactivity or alter ILC2 translational activity and lung pathogenicity depending on the experimental context (Hurrell et al., 2024; Lim et al., 2025). This is exactly the type of setting in which global pharmacologic activation or inhibition is likely to be less informative than cell-resolved approaches (Hellings and Steelant, 2020; Lai and Huang, 2021; Liu et al., 2024).

### 4.4 Acute respiratory distress syndrome and acute lung injury

In acute respiratory distress syndrome and acute lung injury, Piezo1 signaling appears highly sensitive to both cell type and mechanical context. One important study demonstrated that Piezo1 induces apoptosis of type II pneumocytes during acute respiratory distress syndrome, directly linking a mechanosensitive calcium channel to alveolar epithelial cell loss. Subsequent work further connected Piezo1 to mechanical ventilation-associated progression toward fibrosis through epithelial-mesenchymal transition. Together, these findings support the idea that excessive alveolar stretch can push epithelial Piezo1 signaling from physiological sensing into injury amplification (Amado-Rodríguez and Albaiceta, 2014; Spieth et al., 2014; Liang et al., 2019; Fang et al., 2023; Liu et al., 2024).

The endothelial story is more complex. Pressure-induced pulmonary endothelial hyperpermeability has been attributed to Piezo1 signaling, which fits well with pulmonary edema and vascular leak phenotypes. Yet other studies showed that stretch-activated endothelial Piezo1 can relax intrapulmonary arteries and may support adaptive vascular responses. The most reasonable interpretation is not that one set of studies is wrong, but that endothelial Piezo1 behaves differently under microvascular pressure overload, cyclic alveolar stretch, and different phases of acute lung injury (Kása et al., 2015; Lhomme et al., 2019; Lai and Huang, 2021; Wang et al., 2021; Su et al., 2024).

This complexity makes acute lung injury one of the most important disease models for future Piezo1 research. Time-resolved and cell-specific studies could explain how epithelial injury, endothelial leak, and later fibroproliferation are partitioned across the course of disease. Such a framework would be more clinically useful than treating Piezo1 as a static target with a single disease-wide function (Liang et al., 2019; Wang et al., 2021; Fang et al., 2023; Liu et al., 2024).

### 4.5 Respiratory infection and host defense

The infection literature shows that Piezo1 is not only a lung structure sensor but also an immune mechanoreceptor. In innate immunity, PIEZO1 is required for effective mechanosensation of cyclical force and contributes to antibacterial defense programs.

Neutrophil studies further indicate that Piezo1 regulates bactericidal function during transendothelial migration, shear-induced NETosis, proangiogenic specialization in injured lung tissue, and NET-associated macrophage differentiation during influenza virus infection. These studies are especially important in lung disease because they imply that mechanically stressed immune cells use Piezo1 to maintain antimicrobial competence in inflamed tissues (Strieter and Kunkel, 1994; Zhou et al., 2012; Solis et al., 2019; Baratchi et al., 2024; Mukhopadhyay et al., 2024; Wang et al., 2025; Wang et al., 2025).

This creates a major translational caution. In infected lungs, broad Piezo1 inhibition might reduce inflammatory injury in some compartments but simultaneously weaken host defense in neutrophils and other myeloid cells. For this reason, any therapeutic strategy targeting Piezo1 in pneumonia or secondary infection must consider the possibility of trading off tissue protection against microbial clearance (Solis et al., 2019; Mukhopadhyay et al., 2024).

Compared with bacterial infection, direct evidence for Piezo1 in viral respiratory disease remains limited. At present, the more accurate summary is that Piezo1 is mechanistically plausible in virally injured lungs but not yet supported by a disease-specific evidence base comparable to that seen in bacterial defense, pulmonary fibrosis, or pulmonary hypertension (Solis et al., 2019; Liu et al., 2024).

### 4.6 Chronic obstructive pulmonary disease

The evidence linking Piezo1 to chronic obstructive pulmonary disease remains preliminary. Mechanically, chronic obstructive pulmonary disease is a plausible Piezo1-relevant disorder because it features chronic epithelial injury, matrix remodeling, altered airway mechanics, emphysematous loss of elastic recoil, and vascular stress. However, plausibility is not evidence. Current COPD discussions should therefore separate hypothesis generation from disease-specific proof. The most appropriate near-term agenda is to test whether Piezo1 activity tracks epithelial barrier integrity, emphysematous tissue mechanics, pulmonary vascular remodeling, and inflammatory persistence in human COPD samples and mechanistically controlled models. Until those data exist, Piezo1 should not be presented as an established therapeutic target in chronic obstructive pulmonary disease (Barnes, 2017; Dong et al., 2024; Farrell et al., 2024). The major disease- and cell-type-specific roles of Piezo1 discussed above are summarized in **Table 1**.

**Table 1:** Cell-type-specific roles of Piezo1 across respiratory disease contexts.

Cell type	Disease context	Predominant mechanical trigger	Dominant downstream programs	Net biological direction	References
Alveolar epithelial cells	ARDS, ventilator-induced lung injury, post-injury fibrosis	Cyclic stretch, overdistension, epithelial deformation	Calcium influx, apoptosis, ATP release, epithelial-mesenchymal transition	Mainly injurious and profibrotic	(Amado-Rodríguez and Albaiceta, 2014; Spieth et al., 2014; Liang et al., 2019; Fang et al., 2023; Migulina et al., 2023; Liu et al., 2024)
Airway epithelial cells	Asthma, allergic airway disease, barrier dysfunction	Airway narrowing, pressure, epithelial stress, matrix remodeling	Tight junction disruption, barrier leakage, epithelial amplification of inflammation	Mainly barrier disruptive	(Sweerus et al., 2017; Gon and Hashimoto, 2018; Hellings and Steelant, 2020; Lai and Huang, 2021; Bradding et al., 2024; Liang et al., 2024; Liu et al., 2025)
Pulmonary endothelial cells	ALI/ARDS, pulmonary edema, pulmonary hypertension	Shear stress, microvascular pressure, stretch	VE-cadherin and cytoskeletal remodeling, permeability control, nitric oxide signaling, vascular adaptation	Bidirectional and highly context dependent	(Kása et al., 2015; Lhomme et al., 2019; Lai and Huang, 2021; Wang et al., 2021; Su et al., 2024)
Pulmonary arterial smooth muscle cells	Pulmonary hypertension, hypoxic vasoconstriction	Circumferential stretch, altered vascular stiffness, flow-coupled mechanical stress	Phenotypic switching, proliferation, AKT/mTOR and YAP-associated remodeling, vasoreactivity	Mostly pro-remodeling	(Xiao et al., 2020; Liao et al., 2021; Wang et al., 2021; Xu et al., 2022; Yang et al., 2023; Mocumbi et al., 2024; Weinstein et al., 2024)
Fibroblasts and myofibroblasts	IPF, bleomycin fibrosis, ARDS-to-fibrosis transition	Matrix stiffness, cyclic stretch, profibrotic ECM	ERK activation, YAP/TAZ signaling, collagen synthesis, survival and persistence of activated fibroblasts	Strongly profibrotic	(Suki and Bates, 2008; Marinković et al., 2013; Shimbori et al., 2013; Fiore et al., 2015; Júnior et al., 2021; Guo et al., 2022; Trotsyuk et al., 2022; Papavassiliou et al., 2024; Zheng et al., 2024; Xu et al., 2025; Chen et al., 2026)
Myeloid cells, macrophages, neutrophils, and ILC2s	Bacterial lung infection, ALI, inflammatory remodeling	Cyclical force, transendothelial deformation, tissue stiffening, shear stress	Antibacterial defense, inflammatory polarization, NETosis, proangiogenic reprogramming, ILC2 regulation	Protective for host defense but potentially injury-amplifying	(Strieter and Kunkel, 1994; Zhou et al., 2012; Solis et al., 2019; Atcha et al., 2021; Baratchi et al., 2024; Ezzo et al., 2024; Hurrell et al., 2024; Mukhopadhyay et al., 2024; Xie and Hang, 2024; Pirri, 2025; Lim et al., 2025; Wang et al., 2025; Wang et al., 2025; Zhao et al., 2025)

## 5 Translational implications

The central translational lesson from current evidence is that Piezo1 should be viewed as a stratified therapeutic target rather than a universal one. Systemic, nonselective activation or inhibition is unlikely to be optimal because the same intervention could suppress fibrosis in epithelial or fibroblast compartments while impairing protective endothelial signaling or antibacterial immune responses in parallel (Lhomme et al., 2019; Solis et al., 2019; Fang et al., 2023; Mukhopadhyay et al., 2024; Zheng et al., 2024; Xu et al., 2025).

In pulmonary fibrosis, the most promising direction is inhibition of epithelial and fibroblast-lineage Piezo1 during defined profibrotic windows. In pulmonary hypertension, therapeutic design will likely require separate consideration of endothelial and smooth muscle compartments. In acute lung injury, intervention timing may be as important as target cell identity because epithelial apoptosis, endothelial permeability, and later fibroproliferation may not peak simultaneously. In infection, host defense must remain a key constraint (Liang et al., 2019; Liao et al., 2021; Wang et al., 2021; Fang et al., 2023; Mukhopadhyay et al., 2024; Zheng et al., 2024; Xu et al., 2025).

Another important limitation is the current toolset. Existing Piezo1 agonists and inhibitors remain useful for proof-of-concept work, but they do not solve the problem of regional, lineage-specific pulmonary delivery or the risk of opposing effects across epithelial, endothelial, fibroblast, smooth muscle, and immune compartments. For clinical translation, the key question is therefore not simply whether Piezo1 should be activated or inhibited. The key question is how to deliver the correct degree and direction of modulation to the correct cell type during the correct disease window, while preserving host defense and vascular adaptation (Hughes et al., 2005; Migulina et al., 2023; Yang et al., 2023; Beverley and Levitan, 2024; Burgess and Gosens, 2024; Liu et al., 2024; Xiao, 2024; Purali, 2025).

## 6 Knowledge gaps and research priorities

Several major gaps remain. A central unresolved issue is the lack of an integrated framework that combines cell specificity with disease timing. Many studies are designed around a single cell population, which makes it difficult to explain why apparently similar mechanical signals produce divergent outcomes across epithelial, endothelial, smooth muscle, fibroblast, and immune compartments (Bhattacharya and Hough, 2019; Lhomme et al., 2019; Liang et al., 2019; Solis et al., 2019; Liao et al., 2021; Wang et al., 2021; Mukhopadhyay et al., 2024; Zheng et al., 2024).

Human evidence also remains limited and is often cross-sectional. Pulmonary hypertension and pulmonary fibrosis already have supportive evidence from patient-linked tissues and primary cells, but longitudinal human studies connecting Piezo1 activity with imaging, lung mechanics, hemodynamics, and clinical outcomes are still uncommon. This limits the confidence with which mechanistic findings can be translated into therapeutic strategy (Liao et al., 2021; Wang et al., 2021; Zheng et al., 2024; Xu et al., 2025).

The current evidence base is uneven across respiratory diseases. Evidence is strongest in pulmonary fibrosis and pulmonary hypertension, moderate in acute lung injury and asthma, and weak in chronic obstructive pulmonary disease and viral respiratory disease. This imbalance should shape research prioritization and manuscript language. Broad claims about Piezo1 across respiratory disease should be avoided unless they are explicitly qualified by disease, cell type, and whether the cited evidence is direct Piezo1 biology or adjacent mechanobiology.

Pharmacological specificity remains another major barrier. Since Piezo1 can exert opposing effects across cell populations, future studies should prioritize locally delivered, cell-specific interventions and direct head-to-head comparisons of early versus late intervention windows. Acute respiratory distress syndrome-to-fibrosis transition models may be especially informative because they can capture epithelial injury, endothelial leak, immune activation, and fibroproliferation within a single disease continuum (Liang et al., 2019; Wang et al., 2021; Fang et al., 2023; Liu et al., 2024; Mukhopadhyay et al., 2024; Zheng et al., 2024; Xu et al., 2025).

The key translational gaps and corresponding priority study designs are summarized in **Table 2**.

**Table 2:** Translational gaps and priority study designs for Piezo1-targeted research in the lung.

Translational gap	Why it matters	Priority study design	Recommended readouts	References
Lack of unified cell-specific framework	Opposite effects across epithelial, endothelial, smooth muscle, fibroblast, and immune cells complicate drug design	Use lineage-specific knockout or silencing systems in shared disease models	Histology, single-cell RNA-seq, lineage tracing, pathway activation maps	(Bhattacharya and Hough, 2019; Guo et al., 2022; Migulina et al., 2023; Liu et al., 2024)
Poor time resolution across disease phases	Early barrier injury and later fibrosis may require different interventions	Perform time-course studies from acute injury to fibroproliferation	Permeability, apoptosis, BALF ATP, EMT markers, collagen burden, lung mechanics	(Amado-Rodríguez and Albaiceta, 2014; Spieth et al., 2014; Liang et al., 2019; Fang et al., 2023; Liu et al., 2024)
Limited human translational datasets	Most data remain preclinical or cross-sectional	Integrate patient tissues with lung mechanics, imaging, hemodynamics, and outcomes	Spatial transcriptomics, proteomics, clinical phenotyping, organoid validation	(Zhao et al., 2016; Xiao et al., 2020; Wang et al., 2021; Xu et al., 2025)
Insufficient clarity in pulmonary hypertension compartmentalization	Endothelial and smooth muscle Piezo1 may not be therapeutic in the same direction	Direct endothelial versus PASMC comparison in hypoxic and non-hypoxic PH models	Vasoreactivity, right ventricular pressure, vascular muscularization, Notch and YAP signaling	(Xiao et al., 2020; Liao et al., 2021; Wang et al., 2021; Xu et al., 2022; Yang et al., 2023; Mocumbi et al., 2024; Weinstein et al., 2024)
Uncertain infection trade-offs	Broad inhibition may suppress injury but weaken host defense	Test Piezo1 modulation in pneumonia or secondary infection superimposed on ALI	Bacterial clearance, neutrophil killing, NETosis, edema, survival	(Strieter and Kunkel, 1994; Zhou et al., 2012; Solis et al., 2019; Atcha et al., 2021; Baratchi et al., 2024; Ezzo et al., 2024; Hurrell et al., 2024; Mukhopadhyay et al., 2024; Xie and Hang, 2024; Pirri, 2025; Lim et al., 2025; Wang et al., 2025; Wang et al., 2025; Zhao et al., 2025)
Lack of local delivery strategies	Systemic modulators are unlikely to achieve useful precision	Develop inhaled or cell-targeted delivery systems and compare them with systemic dosing	Biodistribution, cell-specific target engagement, efficacy-to-toxicity ratio	(Hughes et al., 2005; Migulina et al., 2023; Yang et al., 2023; Beverley and Levitan, 2024; Burgess and Gosens, 2024; Liu et al., 2024; Xiao, 2024; Purali, 2025)

## 7 Conclusion

Piezo1 has emerged as a central mechanotransduction molecule in respiratory disease, but its significance lies in context dependence rather than uniform biological direction. In the lung, Piezo1 can promote epithelial injury, fibroblast activation, vascular remodeling, and inflammatory amplification, yet in other settings it may support adaptive endothelial signaling or antibacterial defense. The strongest current evidence positions Piezo1 at the center of pulmonary fibrosis and pulmonary hypertension, with increasingly important roles in acute lung injury, airway barrier disease, and mechanical immunity (Lhomme et al., 2019; Liang et al., 2019; Solis et al., 2019; Lai and Huang, 2021; Liao et al., 2021; Wang et al., 2021; Fang et al., 2023; Xu et al., 2022; Yang et al., 2023; Liu et al., 2024; Mukhopadhyay et al., 2024; Zheng et al., 2024; Xu et al., 2025).

The field should now move beyond the simple question of whether Piezo1 is beneficial or harmful. A more informative framework is to ask which pulmonary cell types use Piezo1, under what mechanical conditions, at which disease stage, and toward which downstream program. From a translational perspective, this means that the future of Piezo1-targeted therapy will likely depend on cell-specific, stage-specific, and locally delivered modulation. That model is more consistent with lung biology and more likely to generate clinically meaningful benefit (Li et al., 2014; Li et al., 2015; Bhattacharya and Hough, 2019; Liao et al., 2021; Fang et al., 2023; Liu et al., 2024; Mukhopadhyay et al., 2024; Zheng et al., 2024; Wang et al., 2025; Xu et al., 2025).

## References

[B59] Amado-Rodríguez L. Albaiceta G. M. (2014). Towards prevention of ventilator-induced lung injury: is mechanotransduction the answer? Minerva Anestesiol. 80, 874–876 24430007

[B38] Atcha H. Jairaman A. Holt J. Meli V. Nagalla R. Veerasubramanian P. . (2021). Mechanically activated ion channel Piezo1 modulates macrophage polarization and stiffness sensing. Nat. Commun. 12, 3256. doi: 10.1038/s41467-021-23482-5 34059671 PMC8167181

[B33] Baratchi S. Danish H. Chheang C. Zhou Y. Huang A. Lai A. . (2024). Piezo1 expression in neutrophils regulates shear-induced NETosis. Nat. Commun. 15, 7023. doi: 10.1038/s41467-024-51211-1 39174529 PMC11341855

[B64] Barnes P. J. (2017). Cellular and molecular mechanisms of asthma and COPD. Clin. Sci. (Lond). 131, 1541–1558. doi: 10.1042/CS20160487 28659395

[B21] Beverley K. Levitan I. (2024). Cholesterol regulation of mechanosensitive ion channels. Front. Cell Dev. Biol. 12, 1352259. doi: 10.3389/fcell.2024.1352259 38333595 PMC10850386

[B3] Bhattacharya J. Hough R. (2019). Piezo1 in the lung: at last! Am. J. Respir. Cell Mol. Biol. 60, 609–610. doi: 10.1165/rcmb.2018-0418ED 30653923 PMC6543746

[B56] Bradding P. Porsbjerg C. Côté A. Dahlén S. E. Hallstrand T. S. Brightling C. E. (2024). Airway hyperresponsiveness in asthma: the role of the epithelium. J. Allergy Clin. Immunol. 153, 1181–1193. doi: 10.1016/j.jaci.2024.02.011 38395082

[B16] Burgess J. Gosens R. (2024). Mechanotransduction and the extracellular matrix: key drivers of lung pathologies and drug responsiveness. Biochem. Pharmacol. 228, 116255. doi: 10.1016/j.bcp.2024.116255 38705536

[B49] Chen J. Meng J. Tang X. Liu G. Zhang K. (2026). Unraveling the matrix stiffness landscape in idiopathic pulmonary fibrosis: GSN and ARG1 as novel diagnostic biomarkers and potential therapeutic targets. Int. Immunopharmacol. 174, 116334. doi: 10.1016/j.intimp.2026.116334 41671621

[B4] Coste B. Mathur J. Schmidt M. Earley T. Ranade S. Petrus M. . (2010). Piezo1 and Piezo2 are essential components of distinct mechanically activated cation channels. Science 330, 55–60. doi: 10.1126/science.1193270 20813920 PMC3062430

[B65] Dong L. L. Liu Z. Y. Chen K. J. Li Z. Y. Zhou J. S. Shen H. H. . (2024). The persistent inflammation in COPD: is autoimmunity the core mechanism? Eur. Respir. Rev. 33, 230137. doi: 10.1183/16000617.0137-2023 38537947 PMC10966473

[B37] Ezzo M. Spindler K. Wang J. Lee D. Pecoraro G. Cowen J. . (2024). Acute contact with profibrotic macrophages mechanically activates fibroblasts via αvβ3 integrin-mediated engagement of Piezo1. Sci. Adv. 10, eadp4726. doi: 10.1126/sciadv.adp4726 39441936 PMC11498225

[B17] Fang X. Li M. Wang Y. Zhang P. Sun M. Xu J. . (2023). Mechanosensitive ion channel Piezo1 mediates mechanical ventilation-exacerbated ARDS-associated pulmonary fibrosis. J. Adv. Res. 53, 175–186. doi: 10.1016/j.jare.2022.12.006 36526145 PMC10658225

[B66] Farrell L. A. O’Rourke M. B. Padula M. P. Souza-Fonseca-Guimaraes F. Caramori G. Wark P. A. B. . (2024). The current molecular and cellular landscape of chronic obstructive pulmonary disease (COPD): a review of therapies and efforts towards personalized treatment. Proteomes. 12, 23. doi: 10.3390/proteomes12030023 39189263 PMC11348234

[B45] Fiore V. Strane P. Bryksin A. White E. Hagood J. Barker T. (2015). Conformational coupling of integrin and Thy-1 regulates Fyn priming and fibroblast mechanotransduction. J. Cell Biol. 211, 173–190. doi: 10.1083/jcb.201505007 26459603 PMC4602038

[B5] Ge J. Li W. Zhao Q. Li N. Chen M. Zhi P. . (2015). Architecture of the mammalian mechanosensitive Piezo1 channel. Nature 527, 64–69. doi: 10.1038/nature15247 26390154

[B55] Gon Y. Hashimoto S. (2018). Role of airway epithelial barrier dysfunction in pathogenesis of asthma. Allergol Int. 67, 12–17. doi: 10.1016/j.alit.2017.08.011 28941636

[B15] Guo T. He C. Venado A. Zhou Y. (2022). Extracellular matrix stiffness in lung health and disease. Compr. Physiol. 12, 3523–3558. doi: 10.1002/cphy.c210032 35766837 PMC10088466

[B10] Hellings P. Steelant B. (2020). Epithelial barriers in allergy and asthma. J. Allergy Clin. Immunol. 145, 1499–1509. doi: 10.1016/j.jaci.2020.04.010 32507228 PMC7270816

[B68] Hughes S. El Haj A. J. Dobson J. (2005). Magnetic micro- and nanoparticle mediated activation of mechanosensitive ion channels. Med. Eng. Phys. 27, 754–762. doi: 10.1016/j.medengphy.2005.04.006 15985383

[B39] Hurrell B. Shen S. Li X. Sakano Y. Kazemi M. Quach C. . (2024). Piezo1 channels restrain ILC2s and regulate the development of airway hyperreactivity. J. Exp. Med. 221, e20231835. doi: 10.1084/jem.20231835 38530239 PMC10965393

[B20] Jin P. Jan L. Jan Y. (2020). Mechanosensitive ion channels: structural features relevant to mechanotransduction mechanisms. Annu. Rev. Neurosci. 43, 207–229. doi: 10.1146/annurev-neuro-070918-050509 32084327

[B47] Júnior C. Narciso M. Marhuenda E. Almendros I. Farré R. Navajas D. . (2021). Baseline stiffness modulates the non-linear response to stretch of the extracellular matrix in pulmonary fibrosis. Int. J. Mol. Sci. 22, 12928. doi: 10.3390/ijms222312928 34884731 PMC8657558

[B60] Kása A. Csortos C. Verin A. D. (2015). Cytoskeletal mechanisms regulating vascular endothelial barrier function in response to acute lung injury. Tissue Barriers. 3, e974448. doi: 10.4161/21688370.2014.974448 25838980 PMC4372017

[B13] Lai Y. Huang Y. (2021). Mechanisms of mechanical force induced pulmonary vascular endothelial hyperpermeability. Front. Physiol. 12, 714064. doi: 10.3389/fphys.2021.714064 34671268 PMC8521004

[B24] Lewis A. Grandl J. (2021). Piezo1 ion channels inherently function as independent mechanotransducers. Elife 10, e70988. doi: 10.7554/eLife.70988 34711306 PMC8555984

[B8] Lhomme A. Gilbert G. Pele T. Deweirdt J. Henrion D. Baudrimont I. . (2019). Stretch-activated Piezo1 channel in endothelial cells relaxes mouse intrapulmonary arteries. Am. J. Respir. Cell Mol. Biol. 60, 650–658. doi: 10.1165/rcmb.2018-0197OC 30562052

[B2] Li J. Hou B. Beech D. (2015). Endothelial Piezo1: life depends on it. Channels (Austin) 9, 1–2. doi: 10.4161/19336950.2014.986623 25558961 PMC4594609

[B1] Li J. Hou B. Tumova S. Muraki K. Bruns A. Ludlow M. . (2014). Piezo1 integration of vascular architecture with physiological force. Nature 515, 279–282. doi: 10.1038/nature13701 25119035 PMC4230887

[B7] Liang G. Xu J. Cao L. Zeng X. Chen X. Yang Q. . (2019). Piezo1 induced apoptosis of type II pneumocytes during acute respiratory distress syndrome. Respir. Res. 20, 118. doi: 10.1186/s12931-019-1083-1 31186017 PMC6558715

[B57] Liang J. Zhou C. Zhang C. Liang S. Zhou Z. Zhou Z. . (2024). Nicotinamide mononucleotide attenuates airway epithelial barrier dysfunction via inhibiting SIRT3 SUMOylation in asthma. Int. Immunopharmacol. 127, 111328. doi: 10.1016/j.intimp.2023.111328 38064810

[B11] Liao J. Lu W. Chen Y. Duan X. Zhang C. Luo X. . (2021). Upregulated mechanosensitive channel Piezo1 participates in pulmonary hypertension via the induction of proliferative phenotype in pulmonary arterial smooth muscle cells. Hypertension 77, 1974–1989. doi: 10.1161/HYPERTENSIONAHA.120.16629 33813851

[B40] Lim M. Park S. Joo Y. Kim S. Ham M. Kim T. . (2025). Piezo1-mediated mechanotransduction regulates the translational activity, function and lung pathogenicity of group 2 innate lymphoid cells. Signal. Transduct Target Ther. 10, 269. doi: 10.1038/s41392-025-02350-4 40841361 PMC12370950

[B30] Liu G. Dong B. Devanarayana S. Chen R. Liu Q. (2024). Emerging roles of mechanosensitive ion channels in ventilator induced lung injury: a systematic review. Front. Immunol. 15, 1479230. doi: 10.3389/fimmu.2024.1479230 39664395 PMC11631737

[B53] Liu S. Guo M. Xie X. Zhang X. Zhang X. Xie S. . (2025). Piezo1-induced nasal epithelial barrier dysfunction in allergic rhinitis. Inflammation 48, 2824–2836. doi: 10.1007/s10753-024-02234-9 39798033 PMC12336081

[B44] Marinković A. Liu F. Tschumperlin D. (2013). Matrices of physiologic stiffness potently inactivate idiopathic pulmonary fibrosis fibroblasts. Am. J. Respir. Cell Mol. Biol. 48, 422–430. doi: 10.1165/rcmb.2012-0335OC 23258227 PMC3653602

[B22] Martinac B. (2004). Mechanosensitive ion channels: molecules of mechanotransduction. J. Cell Sci. 117, 2449–2460. doi: 10.1242/jcs.01232 15159450

[B14] Migulina N. Kelley B. Zhang E. Pabelick C. Prakash Y. Vogel E. (2023). Mechanosensitive channels in lung health and disease. Compr. Physiol. 13, 5157–5178. doi: 10.1002/cphy.c230006 37770188

[B50] Mocumbi A. Humbert M. Saxena A. Jing Z. Sliwa K. Thienemann F. . (2024). Pulmonary hypertension. Nat. Rev. Dis. Primers 10, 1. doi: 10.1038/s41572-023-00486-7 38177157

[B23] Morris C. (1990). Mechanosensitive ion channels. J. Membr. Biol. 113, 93–107. doi: 10.1007/BF01872883 1690807

[B28] Mukhopadhyay A. Tsukasaki Y. Chan W. Nguyen H. Hanes R. Hu L. . (2024). Trans-endothelial neutrophil migration activates bactericidal function via Piezo1 mechanosensing. Immunity 57, 52–67.e10. doi: 10.1016/j.immuni.2023.11.007 38091995 PMC10872880

[B48] Papavassiliou K. Sofianidi A. Spiliopoulos F. Gogou V. Gargalionis A. Papavassiliou A. (2024). YAP/TAZ signaling in the pathobiology of pulmonary fibrosis. Cells 13, 1519. doi: 10.3390/cells13181519 39329703 PMC11430237

[B35] Pirri C. (2025). PIEZO channels in mechano-inflammation: gatekeepers of neuroimmune crosstalk. Diseases 13, 263. doi: 10.3390/diseases13080263 40863236 PMC12386134

[B67] Purali N. (2025). Mechanosensitive ion channels: the unending riddle of mechanotransduction. Bioelectricity. 7, 58–70. doi: 10.1089/bioe.2024.0028 40342940 PMC12054614

[B6] Ranade S. Qiu Z. Woo S. Hur S. Murthy S. Cahalan S. . (2014). Piezo1, a mechanically activated ion channel, is required for vascular development in mice. Proc. Natl. Acad. Sci. U.S.A. 111, 10347–10352. doi: 10.1073/pnas.1409233111 24958852 PMC4104881

[B43] Shimbori C. Gauldie J. Kolb M. (2013). Extracellular matrix microenvironment contributes actively to pulmonary fibrosis. Curr. Opin. Pulm. Med. 19, 446–452. doi: 10.1097/MCP.0b013e328363f4de 23872861

[B9] Solis A. Bielecki P. Steach H. Sharma L. Harman C. Yun S. . (2019). Mechanosensation of cyclical force by PIEZO1 is essential for innate immunity. Nature 573, 69–74. doi: 10.1038/s41586-019-1485-8 31435009 PMC6939392

[B58] Spieth P. M. Bluth T. Gama de Abreu M. Bacelis A. Goetz A. E. Kiefmann R. (2014). Mechanotransduction in the lungs. Minerva Anestesiol. 80, 933–941 24299920

[B63] Strieter R. M. Kunkel S. L. (1994). Acute lung injury: the role of cytokines in the elicitation of neutrophils. J. Investig. Med. 42, 640–651 8521027

[B61] Su Y. Lucas R. Fulton D. J. R. Verin A. D. (2024). Mechanisms of pulmonary endothelial barrier dysfunction in acute lung injury and acute respiratory distress syndrome. Chin. Med. J. Pulm. Crit. Care Med. 2, 80–87. doi: 10.1016/j.pccm.2024.04.002 39006829 PMC11242916

[B42] Suki B. Bates J. (2008). Extracellular matrix mechanics in lung parenchymal diseases. Respir. Physiol. Neurobiol. 163, 33–43. doi: 10.1016/j.resp.2008.03.015 18485836 PMC2666313

[B54] Sweerus K. Lachowicz-Scroggins M. Gordon E. LaFemina M. Huang X. Parikh M. . (2017). Claudin-18 deficiency is associated with airway epithelial barrier dysfunction and asthma. J. Allergy Clin. Immunol. 139, 72–81.e1. doi: 10.1016/j.jaci.2016.02.035 27215490 PMC5073041

[B46] Trotsyuk A. Chen K. Hyung S. Ma K. Henn D. Mermin-Bunnell A. . (2022). Inhibiting fibroblast mechanotransduction modulates severity of idiopathic pulmonary fibrosis. Adv. Wound Care (New Rochelle) 11, 511–523. doi: 10.1089/wound.2021.0077 34544267

[B31] Wang J. Zhao W. Bai W. Dong D. Wang H. Qi X. . (2025). PIEZO1 mediates mechanical reprogramming of neutrophils for proangiogenic specialization in the lung. J. Clin. Invest. 135, e183796. doi: 10.1172/JCI183796 40454475 PMC12126238

[B36] Wang Y. Yang Q. Dong Y. Wang L. Zhang Z. Niu R. . (2025). Piezo1-directed neutrophil extracellular traps regulate macrophage differentiation during influenza virus infection. Cell Death Dis. 16, 60. doi: 10.1038/s41419-025-07395-5 39890818 PMC11785962

[B12] Wang Z. Chen J. Babicheva A. Jain P. Suresh K. Rybchyn M. . (2021). Endothelial upregulation of mechanosensitive channel Piezo1 in pulmonary hypertension. Am. J. Physiol. Cell Physiol. 321, C1010–C1027. doi: 10.1152/ajpcell.00147.2021 34669509 PMC8714987

[B52] Weinstein N. Carlsen J. Schulz S. Stapleton T. Henriksen H. H. Travnik E. . (2024). A lifelike guided journey through the pathophysiology of pulmonary hypertension: from measured metabolites to the mechanism of action of drugs. Front. Cardiovasc. Med. 11, 1341145. doi: 10.3389/fcvm.2024.1341145 38845688 PMC11153715

[B18] Xiao B. (2024). Mechanisms of mechanotransduction and physiological roles of PIEZO channels. Nat. Rev. Mol. Cell Biol. 25, 886–903. doi: 10.1038/s41580-024-00773-5 39251883

[B51] Xiao Y. Chen P. P. Zhou R. L. Zhang Y. Tian Z. Zhang S. Y. (2020). Pathological mechanisms and potential therapeutic targets of pulmonary arterial hypertension: a review. Aging Dis. 11, 1623–1639. doi: 10.14336/AD.2020.0111 33269111 PMC7673851

[B32] Xie Y. Hang L. (2024). Mechanical gated ion channel Piezo1: function, and role in macrophage inflammatory response. Innate Immun. 30, 32–39. doi: 10.1177/17534259241249287 38710209 PMC11165660

[B41] Xu L. Li T. Cao Y. He Y. Shao Z. Liu S. . (2025). PIEZO1 mediates periostin-positive myofibroblast activation and pulmonary fibrosis in mice. J. Clin. Invest. 135, e184158. doi: 10.1172/JCI184158 40454481 PMC12126248

[B25] Xu H. Li S. Su M. Wang J. Zhang J. Wang H. . (2022). The role of Piezo1 in pulmonary arterial hypertension: an overview. Front. Cardiovasc. Med. 9, 1021540. doi: 10.3389/fcvm.2022.1021540 36247424 PMC9557227

[B26] Yang Q. Li X. Xing Y. Chen Y. (2023). Piezo1, a novel therapeutic target to treat pulmonary arterial hypertension. Front. Physiol. 14, 1084921. doi: 10.3389/fphys.2023.1084921 36776977 PMC9909334

[B34] Zhao W. Wang J. Wang J. (2025). Mechanotransduction in neutrophil: mechanosensing and immune function regulation. Mechanobiol. Med. 3, 100157. doi: 10.1016/j.mbm.2025.100157 41019411 PMC12466219

[B19] Zhao Q. Wu K. Geng J. Chi S. Wang Y. Zhi P. . (2016). Ion permeation and mechanotransduction mechanisms of mechanosensitive Piezo channels. Neuron 89, 1248–1263. doi: 10.1016/j.neuron.2016.01.046 26924440

[B29] Zheng M. Wang K. Yao Y. Borkar N. Jiang M. Chen H. . (2024). Piezo channels modulate human lung fibroblast function. Am. J. Physiol. Lung Cell. Mol. Physiol. 327, L547–L556. doi: 10.1152/ajplung.00356.2023 39189800 PMC11905809

[B62] Zhou X. Dai Q. Huang X. (2012). Neutrophils in acute lung injury. Front. Biosci. (Landmark Ed). 17, 2278–2283. doi: 10.2741/4051 22652778

